# Trend and treatment status of latent tuberculosis infection patients in Japan – Analysis of Japan TB Surveillance data

**DOI:** 10.1371/journal.pone.0186588

**Published:** 2017-11-01

**Authors:** Lisa Kawatsu, Kazuhiro Uchimura, Akihiro Ohkado

**Affiliations:** Department of Epidemiology and Clinical Research, the Research Institute of Tuberculosis, Japan Anti- tuberculosis Association (RIT/JATA), Tokyo, Japan; Médecins Sans Frontières (MSF), SOUTH AFRICA

## Abstract

**Objective and method:**

Management of latent tuberculosis infection (LTBI) is one of the core elements of End TB Strategy. Japan is one of the few countries in which notification of LTBI is mandatory, yet so far, the data has not been analyzed in detail. We thus conducted a cross-sectional study to examine the trend of LTBI, its treatment outcome and factors predicting treatment non-completion in Japan for the period of 2007 and 2014, using the data from the electronic Japan Tuberculosis Surveillance system. Treatment completion was defined as those whose reason for terminating the treatment was recorded as “treatment completed” and whose treatment duration was 180 days or more.

**Results:**

During the study period, a total of 50,268 LTBI patients were notified, of which 49,525, who had started treatment, were analyzed for characteristics. 57.5% were females, and 38.5% were aged 25–44 years. As for the job category, healthcare professionals, that is medical doctors, nurses and other healthcare workers, consisted the largest group (30.4%). Overall, the number of LTBI notification has been on an increase, with a large increase observed among those aged 65 years and above. Of the 49,525 patients, the information regarding reason for termination of treatment was available for 46,128 (93.1%). Of them, 33,156 (71.9%) had completed treatment as according to the study definition. The risk factors for not completing LTBI treatment included being aged 65 years and above (adjusted odds ratio [aOR] 1.27, 95% confidence interval [95%CI] 1.10–1.47), foreign-born (aOR 1.14, 95%CI 1.02–1.28), healthcare professional (aOR 1.44, 95%CI 1.24–1.69), full-time and part-time worker (aOR 1.40, 95%CI, 1.20–1.63) and detected via contact investigation (aOR 1.26, 95%CI 1.12–1.41).

**Conclusions:**

Our study results revealed that the treatment completion rate was 71.9%, falling short of the national target of 85%, and also that the treatment duration was recorded as less than 180 days for approximately 20% of those who were reported as having completed treatment. Further studies may be built on ours to explore the reasons for not completing the treatment across different population groups, and identify those who benefit the most, and who has the greatest impact on ending TB, by receiving LTBI treatment.

## Introduction

It has been estimated that one third of the world’s population is infected with *M*.*tuberculosis* [1]. In 2015, the World Health Organization (WHO) published a guideline on management of latent tuberculosis infection (LTBI), largely targeted at high- and upper middle-income countries with an estimated tuberculosis (TB) incidence rate of less than 100 per 100,000, in which it recommended active and systematic identification and treatment of people with LTBI for certain high–risk populations [2]. The End TB Strategy also asserts that expansion of preventive treatment for people at high risk is an essential component of the global strategy for TB prevention, care and control beyond 2015 towards the elimination of TB [3].

Japan, which is a TB middle-burden country with a notification of 13.9 per 100, 000 in 2016, is one of the few countries in which notification of LTBI is also made mandatory since 2007. Japan has set a target of 85% and above for completion rate for LTBI treatment [4]. Yet, although the overall trend in the notification of LTBI has been reported annually, its details and especially the treatment status are and have remained unanalyzed. Currently, the electronic Japan TB Surveillance system (JTBS) only has an algorithm to determine the treatment outcome of pulmonary TB at the end of 12 months. However, from the perspective of evaluating the effectiveness of LTBI treatment, including cost-effectiveness, and in guiding policies to decide which population groups to prioritize, analysis of treatment outcome of LTBI is essential. In this study, we thus attempted a detailed analysis of LTBI notifications and on the status of treatment, using the available data from the JTBS.

## Method

The staff of public health centers, who are responsible for receiving TB and LTBI notifications and entering the necessary data to the JTBS, may update the information regarding “treatment status” of the patients every month, until the patient completes his or her treatment, whenever it is deemed necessary. The treatment status is chosen from the following five options– 1. Still on treatment (hospitalized for TB disease), 2.Still on treatment (hospitalize for non-TB disease), 3. Still on treatment (as outpatient), 4. No treatment, and 5. Unknown. When the treatment is terminated, the date, month and year the treatment was terminated, the reason, and the duration of treatment in days, are also entered. Until 2011, the reason for terminating the treatment was chosen from the following four options– 1. Treatment completed, 2. Termination upon physician’s judgement for other reasons, e.g. adverse events, 3. Self-termination, 4. Unknown. In 2012, the JTBS went under a system revision, and the options were increased to nine– 1. Treatment completed, 2. Termination due to adverse events, 3. Termination upon physician’ judgement for reasons other than 1 and 2, 4. Self-termination, 5. Lost to follow-up, 6. Gone back to country of birth (for foreign-born patients), 7. TB dead, 8. Non-TB dead, and 9. Unknown.

In Japan, the Guideline on Treatment of LTBI [5], published by The Prevention Committee and the Treatment Committee of the Japanese Society for Tuberculosis, recommends 6- or 9-months regimen by isoniazid as the first option, followed by the 4- or 6-months regimen by rifampicin, which is only recommended when the possibility of the use of isoniazid is ruled out. Initial analysis of the JTBS data indicated that 95% of LTBI patients notified between 2007 and 2014 had received isoniazid monotherapy, and also that the proportion had not decreased during the study period (i.e. the proportions of those receiving isoniazid monotherapy was 93.7% in 2007 and 95.4% in 2014). For the purpose of our study, we thus assumed that the proportion of those receiving shorter regimens, such as 4- or 6-months by rifampicin, has constantly remained minimal, and defined “LTBI treatment completed” as those whose reason for terminating the treatment was “treatment completed”, and whose duration of treatment was 180 days or longer, and all others as “LTBI treatment not completed”. We analyzed the cohort data of those notified as LTBI between 2007 and 2014, to examine the treatment status at the end of 12 month by basic demographic characteristics, and also conducted multiple regression analysis to identify factors contributing to non-completion of LTBI treatment. Using the data from 2012 onward, from which more details are available, we further analyzed the reasons for not completing the LTBI treatment, among those high risk groups identified from the multiple regression analysis.

R version 3.1.3 (R Development Core Team, Vienna, Austria) was used for all statistical analyses. Ethical clearance was not required as the JTBS data does not include case identifiers, as according to the Ethical Guidelines for Epidemiological Research established by Ministry of Education, Culture, Sports, Science and Technology and Ministry of Health, Labour and Welfare of Japan.

## Results

### Characteristic and the trend of newly notified LTBI patients

**Fig 1:**
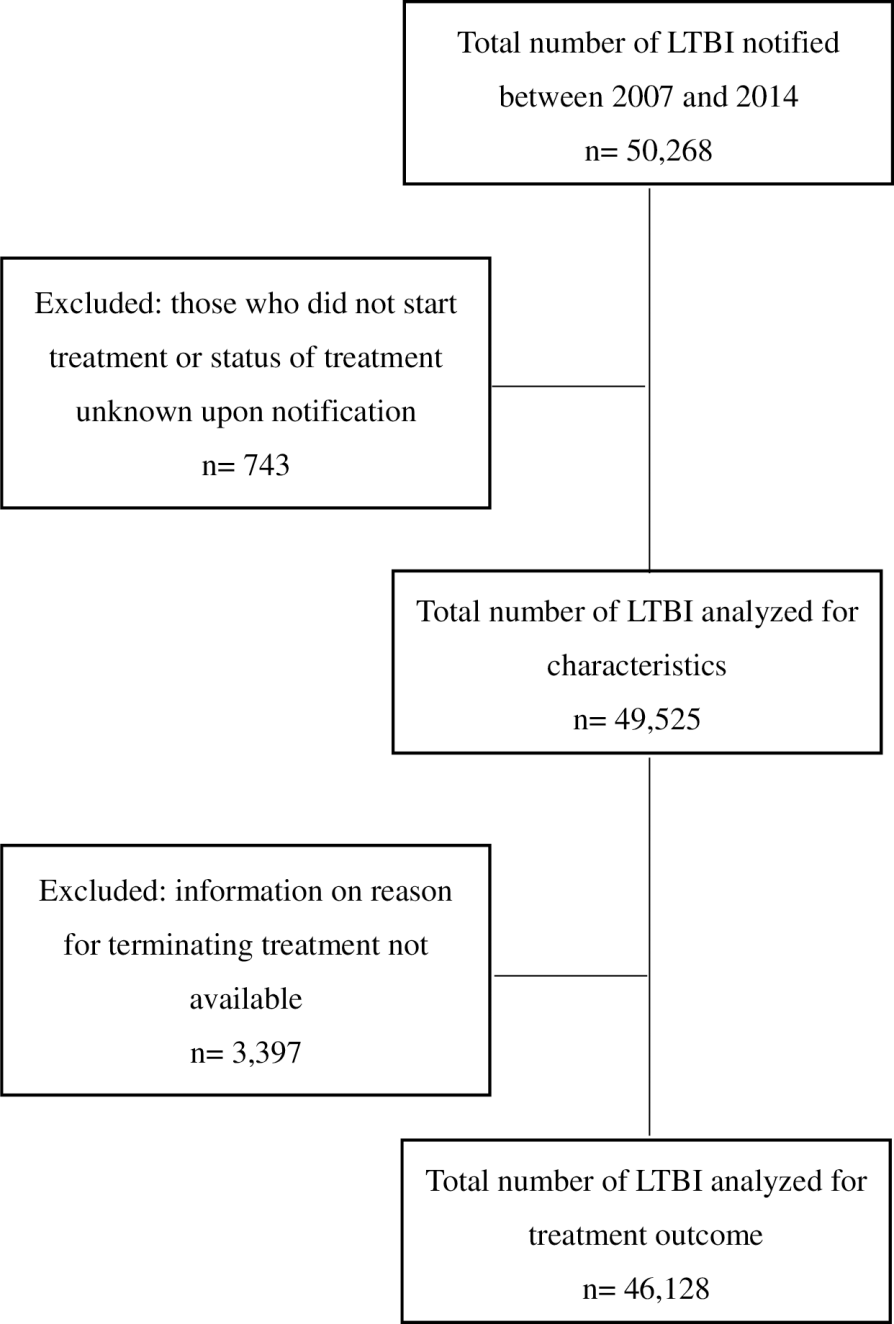
Flow chart of LTBI analyzed in this study.

Flow chart of LTBI patients analyzed in this study is shown in Fig 1. Between 2007 and 2014, a cumulative total of 50,268 LTBI patients were notified to the JTBS, of which 743 (1.5%) either did not initiate treatment or whose treatment status was unknown upon notification. These were considered as initial lost to follow-up and were excluded from the analysis. Characteristics of the 49,525 patients are summarized in Table 1.

**Table 1:** Characteristics of newly notified LTBI patients who initiated treatment in Japan, 2007–2014.

	n	%
**Total**	49,525	100.0
**Sex**		
Male	21,059	42.6
Female	28,466	57.5
**Age group (years)**		
0–24	12,730	25.7
25–44	19,058	38.5
45–64	13,709	27.7
65+	4,028	8.1
**Country of birth**		
Japan-born	45,581	92.0
Foreign-born	2,844	5.8
Unknown	1,100	2.2
**Job category**		
Healthcare professional	15,064	30.4
Full-and part-time worker	13,550	27.4
Temporary worker and unemployed	9,392	19.0
Infants and pre-school children	4,205	8.5
High school and university student	2,744	5.5
Primary school student	2,629	5.3
Housemaker	1,227	2.5
Unknown	714	1.4
**Mode of detection**		
Contact investigation	38,780	78.3
Via routine work, school and community screening	3,263	6.6
Hospitalized for, or via medical check-up for non-TB	2,322	4.7
Via seeking medical attention	2,219	4.5
Unknown	1,129	2.3
Via mass screening for specific population	979	2.0
Via personalized medical check-up	811	1.6
During follow-up for TB	22	0.0

57.5% were females, and 38.5% were aged 25–44 years. 92.0% were Japan-born. As for the job category, healthcare professionals, that is medical doctors, nurses and other healthcare workers, consisted the largest group (30.4%), followed by other full-time and part-time workers (27.4%). 78.3% were detected via contact investigation.

**Fig 2:**
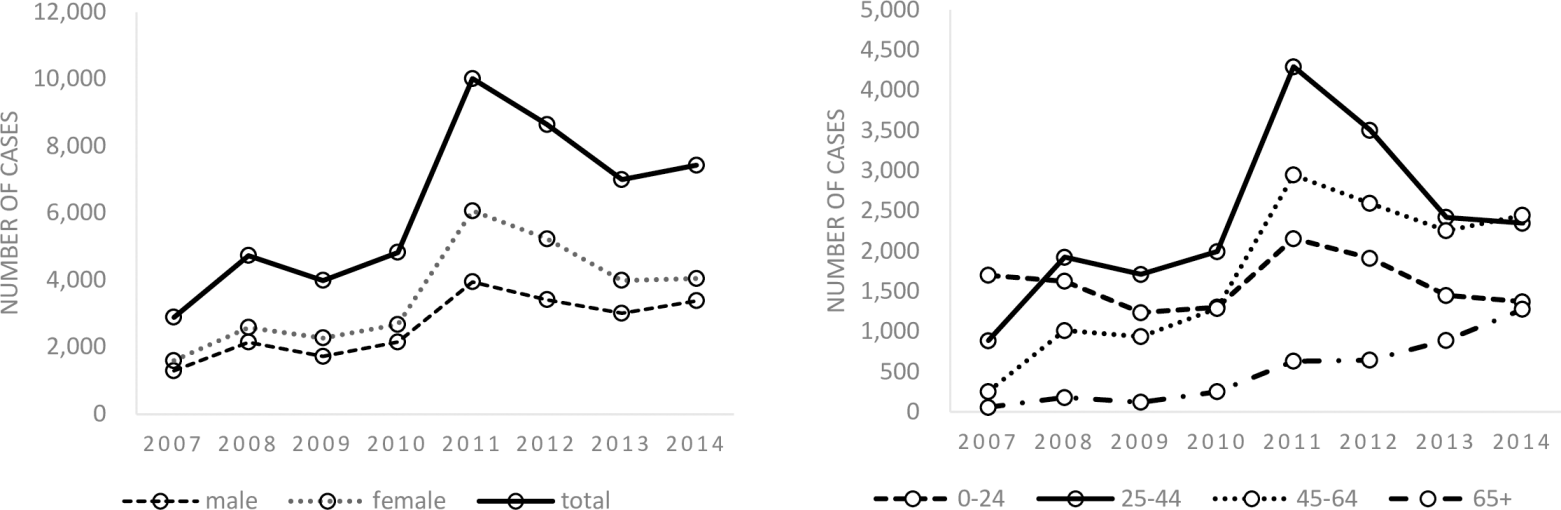
2A and 2B. LTBI notification in Japan, by sex (2a) and age group (2b), 2007–2014.

**Fig 3:**
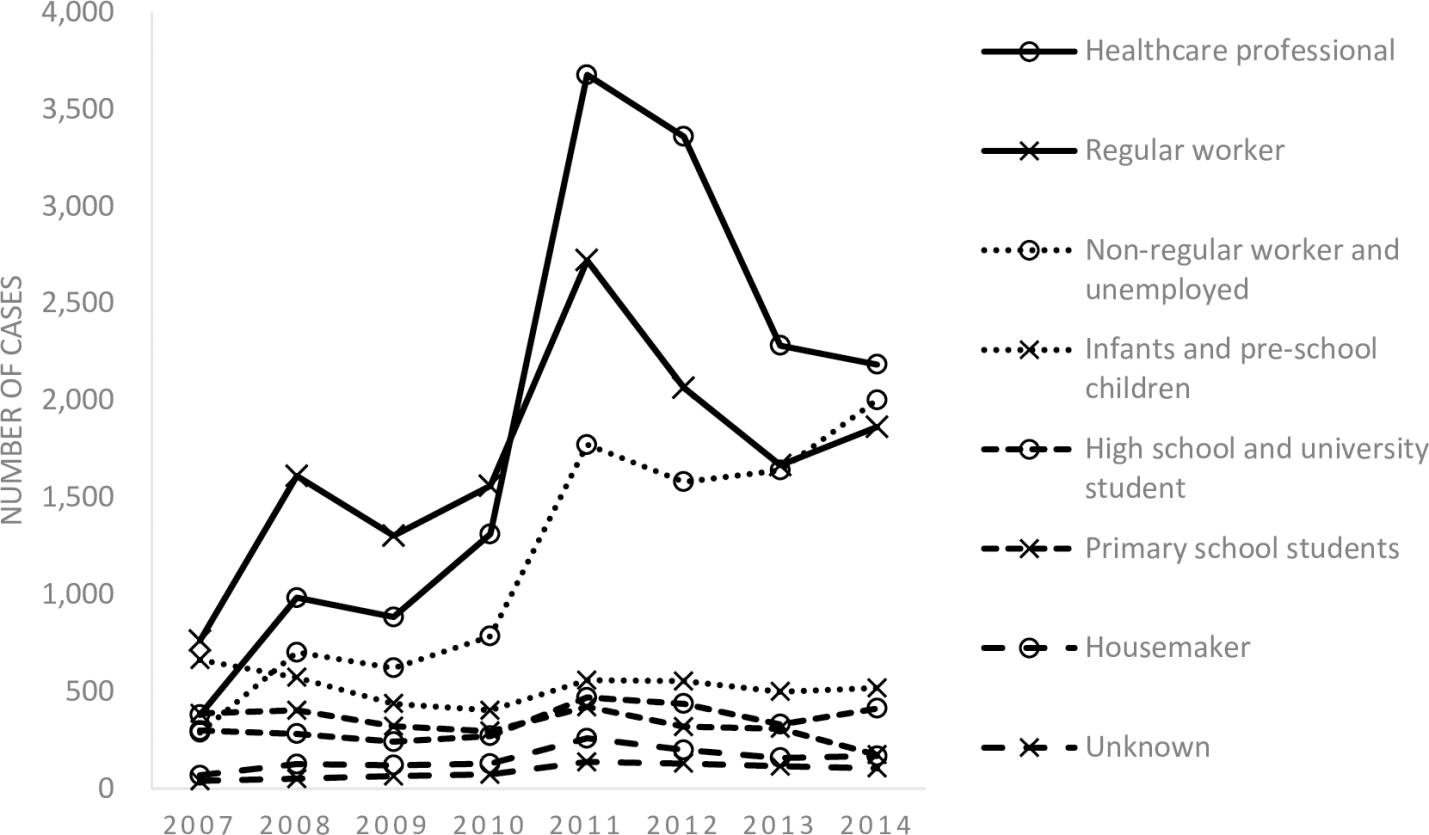
LTBI notification by job category, 2007–2014.

**Fig 4:**
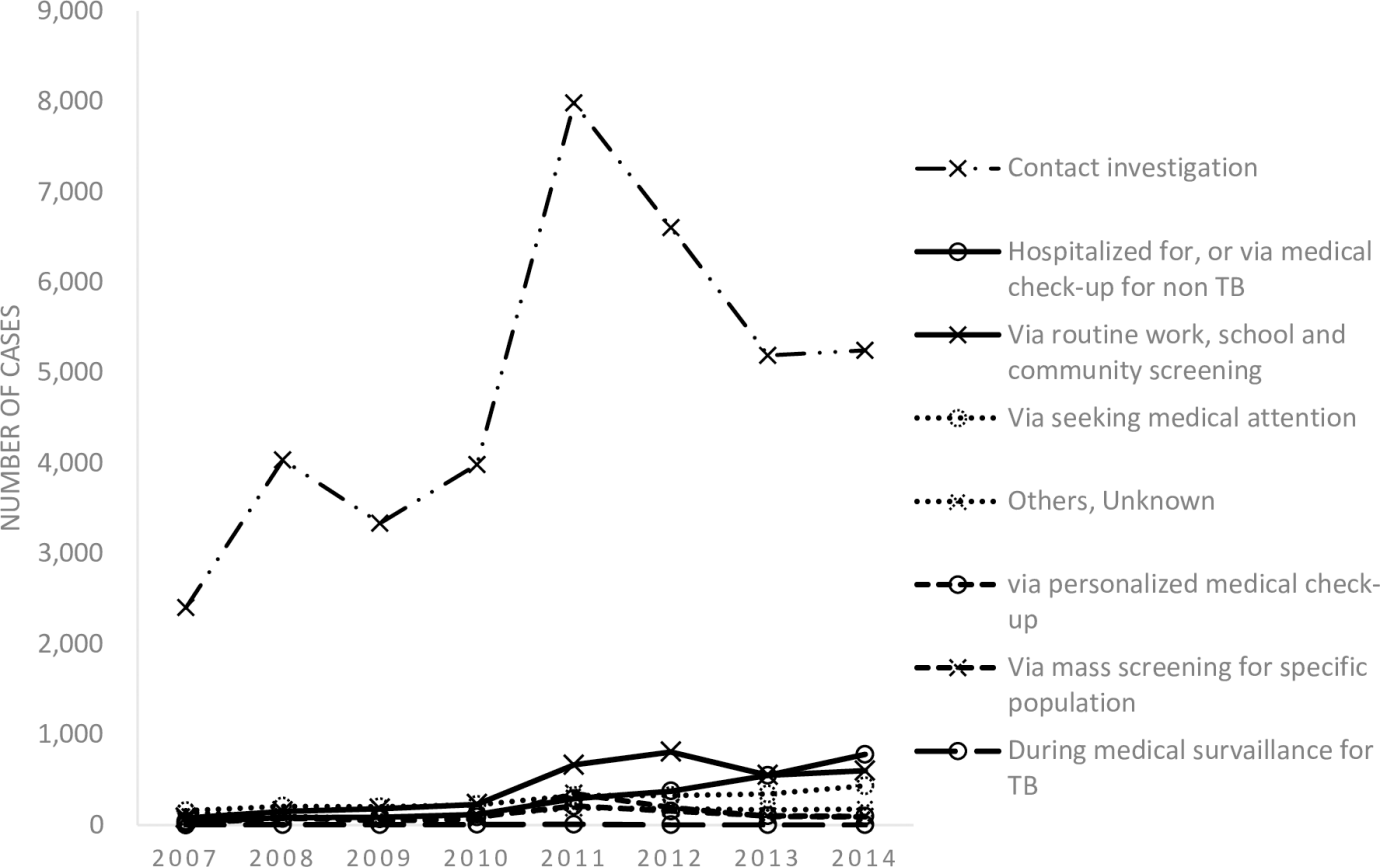
LTBI notification by mode of detection, 2007–2014.

The trend in the notification by sex and age groups is shown in Fig 2A and 2B (see also S1 and S2 Tables). Overall, the number of LTBI notification has been on an increase between 2007 and 2014, with notification among females constantly being slightly above that among males. Looking at the notification trend by age groups, the number has increased in all age groups except the age group 0 to 24 years old. The largest increase was observed among those aged 65 years and above, where the number of notification increased by 22 times, from 55 in 2007 to 1,274 in 2014. The increase in the notification was similarly observed among both Japan- and foreign-born patients (see S3 Table). Trend in the notification by job category and the mode of detection is shown in Figs 3 and 4 (see also S4 and S5 Tables). The numbers of healthcare professionals, full-time and part-time workers and temporary-workers, self-employed and unemployed, have significantly increased while others remained constant over the study period (Fig 3). In terms of the mode of detection, while the number of those detected via contact investigation continued to increase, the largest increase was observed among those detected while being hospitalized for, or during a medical check-up for diseases other than TB, where the number increased by 20 times, from 40 in 2007 to 780 in 2015 (Fig 4).

### LTBI treatment completion rate

Of the 49,525 notified patients who initiated treatment, the information regarding reason for termination of treatment was available for 46,128 patients (93.1%). Of them, 41,143 (89.2%) had a record of “treatment completed” as their reason for terminating their treatment, yet in fact, only 33,156 (71.9%) had fulfilled our definition of “completed treatment”. Table 2 compares the characteristics of the cumulative total of LTBI patients newly notified in Japan between 2007 and 2014 (n = 46,128), by their treatment status as according to the study definition.

**Table 2:** Characteristics of a cumulative total of LTBI patients notified in Japan between 2007 and 2014 who initiated treatment, by their treatment status.

	Not completed		Completed		
	n	%	n	%	p-value
**Total**	12,972	28.1	33,156	71.9	
**Sex**					0.916
Male	5,387	27.7	14,043	72.3	
Female	7,585	28.4	19,113	71.6	
**Age group (years)**					<0.001
0–24	3,128	26.4	8,710	73.6	
25–44	4,889	27.7	12,768	72.3	
45–64	3,835	29.7	9,096	70.3	
65+	1,120	30.3	2,582	69.7	
**Country of birth**					0.004
Japan-born	11,984	28.1	30,623	71.9	
Foreign-born	772	30.8	1,738	69.2	
Unknown	216	21.4	795	78.7	
**Job category**					<0.001
Primary school student	594	23.7	1,912	76.3	
Infants and pre-school children	1,002	25.5	2,928	74.5	
Housemaker	311	27.0	840	73.0	
High school and university student	672	27.1	1,805	72.9	
Temporary worker and unemployed	2,423	28.1	6,200	71.9	
Healthcare professional	4,098	28.9	10,081	71.1	
Full-time and part-time worker	3,682	29.2	8,945	70.8	
Unknown	190	29.9	445	70.1	
**Mode of detection**					<0.001
Via routine work, school and community screening	754	24.9	2,278	75.1	
Hospitalized for, or via medical check-up for non-TB	534	25.6	1,553	74.4	
Via mass screening for specific population	239	26.4	665	73.6	
Via personalized medical check-up	195	26.9	529	73.1	
Via seeking medical attention	593	29.6	1,412	70.4	
Contact investigation	10,310	28.4	26,016	71.6	
Unknown	340	32.9	694	67.1	
During follow-up for TB	7	43.8	9	56.3	

There were no statistically significant difference by sex (*p* = 0.916), however, the completion rate varied among the different age groups (*p*<0.001). The treatment completion rate also differed by the country of birth, with that among the Japan-born significantly higher than among the foreign-born patients (*p* = 0.004). In terms of job category, the treatment completion rate significantly differed, with the highest rate observed among the primary school student (76.3%) and the lowest among those whose job status was unknown (70.1%), followed by healthcare professionals (71.1%). The treatment completion rate was the lowest among those detected during follow-up for TB (56.3%), and the highest among those detected via routine screening at workplace, school or community (75.1%).

**Fig 5:**
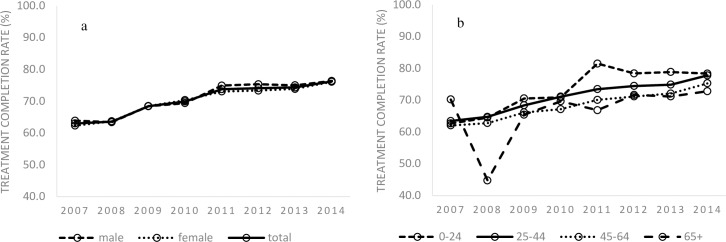
5A and 5B. LTBI completion rate in Japan, by sex (5a) and age group (5b), 2007–2014.

Over the study period, the overall treatment completion rate has steadily increased, from 63.0% in 2007 to 76.2% in 2014, and for both sexes and all age groups (Fig 5A and 5B, see also S6 and S7 Tables). The upward trend was similarly observed regardless of the country of birth, job category, and mode of detection (see S8–S10 Tables).

Table 3 summarizes the results of a multiple regression analysis for factors predicting non completion of LTBI treatment.

**Table 3:** Adjusted odds ratios from multiple logistic regression predicting non-completion of LTBI treatment, in Japan, registered between 2007 and 2014.

	Adjusted OR	95%CI	p-value
Sex			
Female	1.00	0.94–1.06	0.968
**Age group (years)**			
0–24	Reference		
25–44	0.93	0.84–1.03	0.14
45–64	1.07	0.96–1.19	0.233
65+	1.27	1.10–1.47	<0.001
**Country of birth**			
Japan-born	Reference		
Foreign-born	1.14	1.02–1.28	0.024
**Health insurance**			
Covered	Reference		
Not covered	1.56	0.65–3.74	0.321
**Current or history of homelessness**			
No	Reference		
Yes	0.77	0.53–1.12	0.169
**Job category**			
Primary-school student	Reference		
Healthcare professional	1.44	1.24–1.69	<0.001
Unknown	1.42	1.04–1.92	0.026
Full-time and part-time worker	1.40	1.20–1.63	<0.001
Housemaker	1.35	1.08–1.69	0.007
Temporary worker and unemployed	1.24	1.05–1.45	0.011
High-school and university students	1.22	1.04–1.44	0.016
Infants and pre-school children	1.22	1.05–1.42	0.008
**Mode of detection**			
Via routine work, school and community screening	Reference		
Unknown	1.78	1.45–2.19	<0.001
Via seeking medical attention	1.40	1.18–1.66	<0.001
Contact investigation	1.26	1.12–1.41	<0.001
Via personalized medical check-up	1.18	0.93–1.49	0.181
During follow-up for TB	2.88	0.83–10.02	0.097
Hospitalized for, or via medical check-up for non-TB	0.98	0.82–1.17	0.851
Via mass screening for specific population	0.94	0.75–1.19	0.632

The risk factors for not completing LTBI treatment were being aged 65 years and above, all job categories with primary-school students as reference, mode of detection unknown, being detected upon seeking medical attention, and being detected through contact investigation.

**Fig 6:**
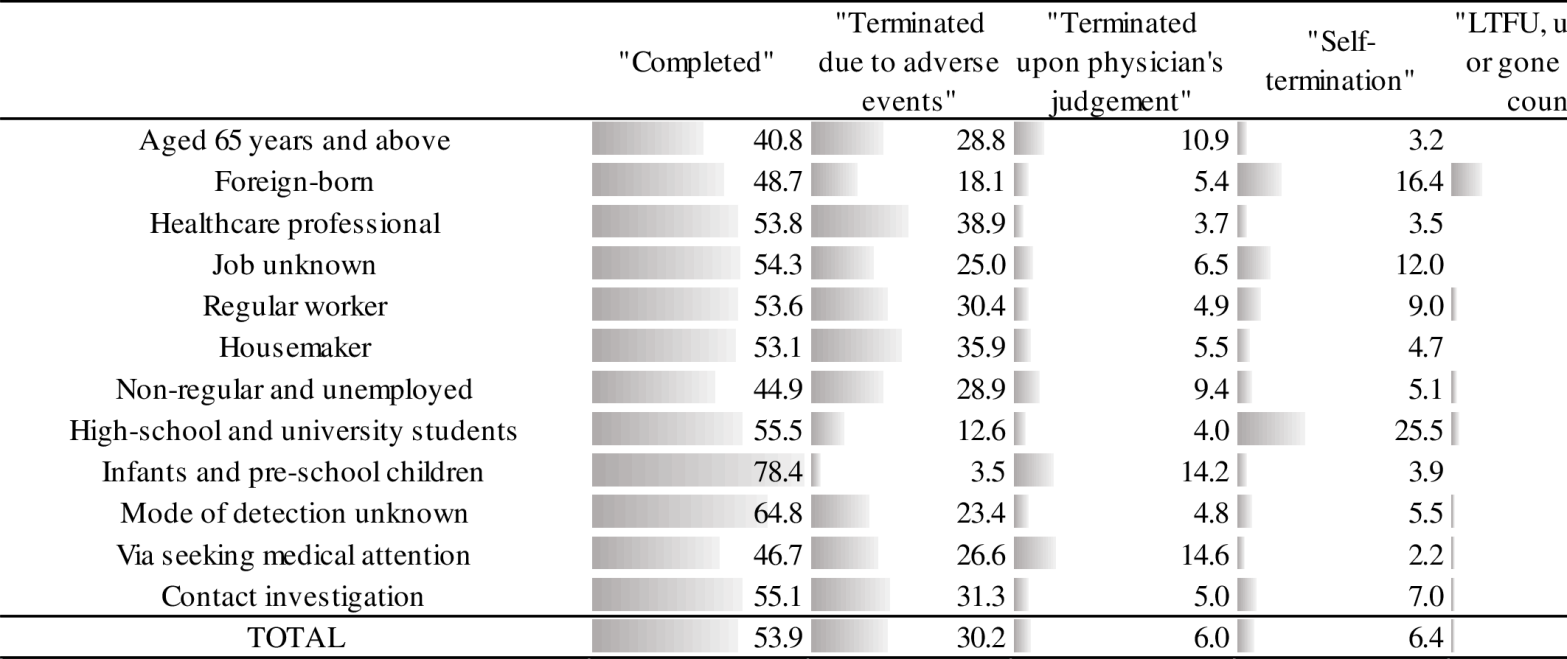
Reasons for terminating LTBI treatment among selected population groups, 2012–2014. The figure shows reasons for terminating LTBI treatment among those who did not meet the criteria of “treatment completion” of our study, stratified by their risk group. TOTAL: The total is not the sum of the specific population groups listed above, but is the total number those who did not meet the criteria of the study definition of “treatment completion”. “Completed”: The physician has judged that the treatment has been completed, despite treatment duration not reaching 180 days. “Terminated upon physician’s judgement”: the physician has terminated the treatment for reasons other than treatment completion and adverse events. “LTFU”: Lost to follow-up.

**Fig 7:**
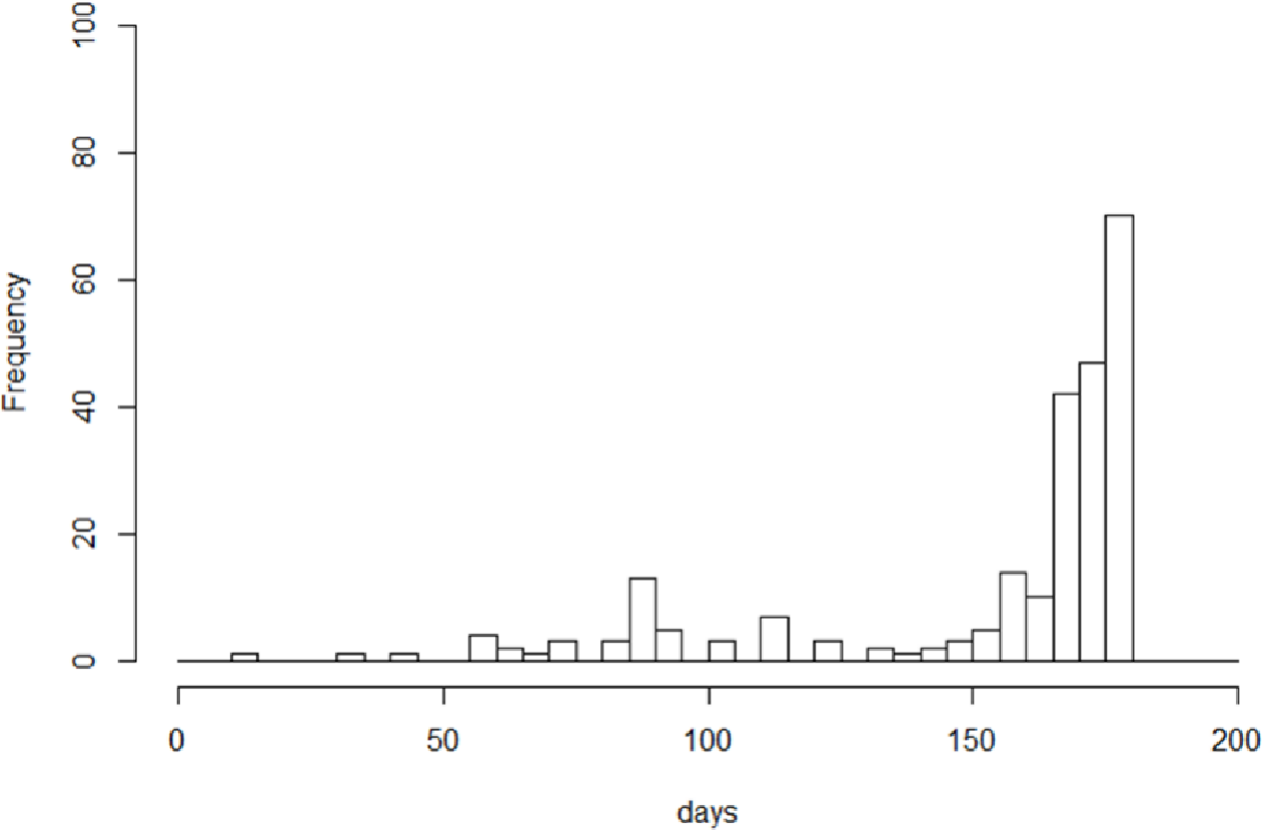
Treatment duration (in days) of infants and pre-school children who did not complete treatment, 2012–2014. The figure shows the treatment duration, in days, of infants and pre-school children who did not complete the treatment according to the study definition but were nevertheless recorded as “treatment completion”.

Fig 6 shows the reasons for terminating LTBI treatment among those who did not meet the criteria of “treatment completion” of our study, stratified by their risk group (see also S11 Table). For all groups, approximately half had terminated the treatment due to the physician judgment that the treatment had been completed despite the treatment duration not reaching 180 days (“completed”)–for infants and pre-school children, the percentage was as high as 78.4%. A sub-analysis of the treatment duration of these infants and pre-school children indicated that approximately half had terminated the treatment after 160 days (Fig 7). The proportion of those who had terminated treatment because of adverse events was the highest among healthcare professionals (38.9%). The proportion of those who had terminated treatment upon physician judgement, for reasons other than adverse events and completion (“terminated upon physician’s judgement”), was the highest among those who were diagnosed via seeking medical attention (14.6%), followed by infants and pre-school children (14.2%). The proportion of those who had terminated upon his or her own decision was the highest among high-school and university students (25.5%), followed by foreign-born (16.4%). The proportion of those either lost to follow-up, whose reason for non-completion was unknown, or who had gone back to their country was the highest among the foreign-born (10.5%), and those who had died was the highest among those aged 65 years and above (16.3%).

## Discussion

In overall, the number of LTBI notification in Japan has steadily increased–to a certain extent, this may reflect the change in the Infectious Diseases Control Law regarding public subsidy for LTBI, which came into effect in 2007. Prior to 2007, the cost of LTBI testing and treatment was only subsidized for those aged 29 years and below (in other words, those considered likely to be infected recently). However, with the increasing evidence to support the effectiveness of LTBI treatment for those with risk factors other than recent infection, the age limit was abolished in 2007 and all notified LTBI cases became eligible for public subsidy [4]. This explains the increase in the number of LTBI notification among those aged between 25 and 64 years, and in terms of job category, among those with jobs but not among students and children. The spreading of the recognition about the benefits of LTBI treatment for those with clinical risk factors, such as immunosuppressive therapy and use of biological agents, among the general practitioners has also likely had the effect of increasing the number of those detected while being hospitalized for, or via medical check-up for diseases other than TB. On the other hand, the sharp increase in notifications in 2011 in those detected via contact investigation between 2011 and 2012 is most likely due to the change in the guideline for contact investigation in 2010, which, following the abolishment of age limit for public subsidy, removed the age limit for testing for LTBI [6]. The reasons for the sharp decline between 2011 and 2012 are not clear, however, Ohkado and colleagues speculate excessive IGRA testing following the abolishment of age limit, and gradual normalization in the following years [6].

As for the treatment completion rates, to our knowledge, this is the first attempt at analyzing the national surveillance data to determine the treatment status of notified LTBI patients in Japan. Kasai and colleagues have reported the treatment outcome of LTBI patients notified in Osaka city between 2011 and 2013, using the same definitions used for the cohort analysis of pulmonary TB patients, and have reported the proportion of treatment completed at 85.4%, lost to follow-up at 12.2%, died at 0.4%, and transferred out at 1.9% (the treatment outcomes recalculated by the authors to exclude those who did not initiate treatment from the denominator) [7]. Their treatment completion rate was slightly higher than our estimate for the same period, which was 74.0%. There have also been several studies from abroad which have reported treatment outcome of LTBI with varying completion rates for specific population groups such as contacts [8–11], foreign-born [12,13], HIV-positive patients [14], injection drug users [15], and jail inmates [16],–however, considering the heterogeneity of the background of these studies, it is probably not meaningful to make a cross-country comparison of LTBI treatment completion rates.

Numerous studies have also examined the factors predicting adherence to LTBI treatment. In general, demographic factors such as age, sex and country of birth do not appear to influence adherence. Although in our study, old age was a risk for non-completion, most likely due to adverse events but also death, other studies have reported otherwise, for example with age > = 65 years [17], >35years [18] and increasing age [19] being associated with better outcomes. Sex and country of birth have also varied in direction–while not a significant factor in our study, higher completion rate was reported among females in some studies [18, 20], while vice versa in others [21]. Being foreign-born was a predicting factor of non-completion of treatment in our study, however, two studies from the US found that the completion rate was better among the foreign-born population [17, 18]. The analysis of the reasons for terminating LTBI treatment in our study indicated that the proportions of self-termination and lost to follow-up (including returning to own country) were high among the foreign-born. This may indicate the need to strengthen culturally and linguistically sensitive patient education and adherence support. The two indicators of socio-economic status, namely the status of health insurance and history of or current homelessness, were not significant factors in our study. The results from previous studies have also been inconsistent, with lack of insurance and homelessness being associated with poor adherence in some studies [18, 19], while vice versa in others [19]. This to a certain extent is dependent on the availability of adherence support for populations considered to be at a higher risk of becoming lost to follow-up. In countries where people such as homeless receive intense assistance, including Japan, their treatment completion rate is often no different to, or better than the general population [16].

As for job category, all job categories were at a higher risk for non-completion, with primary school children as reference. Several other studies have reported poor adherence among healthcare workers [22, 23], however, reasons are not explored. In our study, the proportion of those who had terminated LTBI treatment due to adverse events was especially high among healthcare professionals. As there are no evidence to suggest that the occurrence of adverse events is higher among healthcare workers compared with those in other professions, this may suggest a possibility of the former being more conscious of, due to their expertise, and reacting more frequently, to adverse events. As for other workers, the biggest difference with the primary school children is probably the fact that the former receives far less routine supervision and support for medication than school children, who obviously are being taken cared of both by their parents and school. On the other hand, however, compared with the primary school children, infants and pre-school children had slightly but significantly higher rates of non-completion. The proportion of those terminating the treatment due to the physician’s judgement that the treatment is complete, despite not reaching 180 days, was extremely high among the infants and pre-school children (78.4%), however, approximately half had in fact continued the treatment until 160 days. The general difficulty faced by parents and physicians in administering medication to very small children may encourage early termination of treatment (yet with the perception that the treatment was “sufficient”). A further study may be conducted to explore the circumstances surrounding LTBI treatment for infants.

In terms of the mode of detection, it was puzzling to find those diagnosed as LTBI while seeking medical attention in the first place (2,005 out of the 46,128 analyzed for treatment outcome). This usually describes patients with active TB, who were diagnosed upon seeking medical attention with symptoms suggestive of TB. Since LTBI patients do not present such symptoms, this group may have resulted out of misclassification upon entering data. Those diagnosed via contact investigation was also identified as being at a higher risk of non-completion. Our results are in agreement with a previous study conducted by Izumi and colleagues, who have evaluated contact investigation practices of several public health centers in Japan [24]. The outcome varied considerably across the public health centers, indicating to the possible differences in the performance of public health centers influencing LTBI treatment outcome–for example, those public health centers which conduct highly selective LTBI screening, focusing mainly on close contacts, may have better LTBI treatment outcome among their patients, and vice versa. Our results may therefore point to the need not only in strengthening patient education but also to revise the very selection process itself, of who, among the contacts, are truly at risk of progressing to active TB in accordance with the contact investigation guideline [25]. Finally, those whose job category and mode of detection were “unknown” were predictive factors for non-completion–the most likely explanation is that these represent a case whereby the public health center nurse could not even make the initial contacts to collect such basic information. In other words, these may be considered equivalent to “quasi” lost to follow-up where the patient may agree to initiate treatment at first but fails to continue very early in the course of the treatment.

This study is not without limitations. Firstly, we determined the treatment outcome from data regarding “reasons for terminating LTBI treatment” of the JTBS. Unlike the treatment outcome for pulmonary TB, which is determined by a computerized algorithm, the reasons for terminating LTBI treatment are simply chosen and entered by the staff of public health centers from a pull-down menu of the JTBS system, as according to what is recorded on the patient registration card. Though we attempted to validate “treatment completed” by examining the treatment duration, there were no means to validate other reasons. There is thus the possibility of misclassification for reasons other than “treatment completed”. Secondly, we were not able to consider the effect of shorter treatment regimen, which, in numerous previous studies, has been associated with better completion of LTBI treatment [26–28]. However, as the current situation in Japan is that the majority of the LTBI regimen registered is either 6 or 9 months of isoniazid, it may be reasonable to assume that the effect of shorter regimen e.g. 4 months of rifampicin, is still minimal. Thirdly, as we could only collect information regarding the initial treatment regimen, we were unable to consider the effect of those who had changed regimen during the course of their treatment. Lastly, investigation into the effect of perceptions and beliefs of the patients was beyond the scope of this study.

## Conclusions

Despite management of LTBI being identified as one of the critical components of End TB Strategy, our study results revealed that the treatment completion rate, that is those who had completed the recommended 6 months therapy of isoniazid, was 71.9%, falling short of the national target, and also that the treatment duration of approximately 20% of those who had been recorded as “treatment completed” had in fact terminated treatment early. Further studies are needed to explore the reasons behind the “treatment completed, yet treatment duration falling short of 180 days”. Other various reasons were identified for terminating the treatment across different populations–these may point to the need for tailor-made interventions to improve adherence. However, just as important is the need to critically assess the risk of developing active TB for different population groups, and identify those who benefit the most, and who has the greatest impact on ending TB, by receiving LTBI treatment.

## Supporting information

S1 Table LTBI notification by sex, 2007–2014. (XLSX)

S2 Table LTBI notification by age group (years), 2007–2014. (XLSX)

S3 Table LTBI notification by country of birth, 2007–2014. (XLSX)

S4 Table LTBI notification by job category, 2007–2014. (XLSX)

S5 Table LTBI notification by mode of detection, 2007–2014. (XLSX)

S6 Table LTBI treatment outcome, by year of notification and sex. (XLSX)

S7 Table LTBI treatment outcome, by year of notification and age group (years). (XLSX)

S8 Table LTBI treatment outcome, by year of notification and country of birth. (XLSX)

S9 Table LTBI treatment outcome, by year of notification and job category. (XLSX)

S10 Table LTBI treatment outcome, by year of notification and mode of detection. (XLSX)

S11 Table Reasons for terminating treatment among LTBI patients who did not meet the study definition of "treatment. (XLSX)
